# *C. quinoa* and *S. hispanica* L. Seeds Provide Immunonutritional Agonists to Selectively Polarize Macrophages

**DOI:** 10.3390/cells9030593

**Published:** 2020-03-02

**Authors:** Maša Srdić, Ivana Ovčina, Bartosz Fotschki, Claudia Monika Haros, Jose Moises Laparra Llopis

**Affiliations:** 1 Madrid Institute for Advanced Studies in Food (IMDEA Food). Ctra. Cantoblanco 8, 28049 Madrid, Spain; masa.srdic@pbf.hr (M.S.); ivanaovcina@gmail.com (I.O.); 2 Department of Biological Function of Food, Institute of Animal Reproduction and Food Research, Polish Academy of Sciences, Tuwima 10, 10-748 Olsztyn, Poland; bartosz_fotschki@wp.pl; 3 Instituto de Agroquímica y Tecnología de Alimentos (IATA), Consejo Superior de Investigaciones Científicas (CSIC), Av. Agustín Escardino 7, Parque Científico, 46980 Paterna, Valencia, Spain; cmharos@iata.csic.es

**Keywords:** innate biology, immunotherapy, natural products, cancer chemoprevention

## Abstract

Diet-related immunometabolic-based diseases are associated with chronic inflammation in metabolic tissues, and infiltrated macrophages have been suggested as mediators for tissue- damaging inflammation. Growing evidence implicates *Chenopodium quinoa* and *Salvia hispanica* L. as important contributors to immunonutritional health. However, the functional roles of the immunonutritional protease inhibitors (PPIs) found in these crops on the macrophages’ metabolic and phenotypic adaptation remain to be elucidated. The salt soluble fraction of proteins was extracted and analyzed confirming the presence of 11S and 2S albumin. The <30 kDa fraction of the extract from both crops was subjected to simulated gastrointestinal digestion, where (RP-LC-MS/MS analyses) polypeptides from 2S-type of proteins were found, along with the 2S albumin (13 kDa) for *S. hispanica* in the bioaccessible fraction (BAF). Using human-like macrophage cells to deepen our understanding of the modulatory effects of this BAF, FACS analyses revealed their potential as TLR4 agonists, favoring increased phenotypic CD68/CD206 ratios. The results of mitochondrial stress tests showed that cells increased oxygen consumption rates and non-mitochondrial respiration, confirming negligible deleterious effects on mitochondrial function. At molecular-level, adaptation responses shed light on changes showing biological correlation with TLR4 signaling. The resulting immunometabolic effects triggered by PPIs can be a part of a tailored nutritional intervention strategy in immunometabolic-based diseases.

## 1. Introduction

Western diets, due to their characteristic energy distribution, significantly disrupt immune signals that stem at the intestinal level, thus causing imbalances in the environment-intestinal-immune interactions that can favor the appearance of diverse metabolic alterations such as obesity, type 2 diabetes and metabolic syndrome [1,2]. These represent important risk factors for non-alcoholic fatty liver disease (NAFLD). Alterations in liver function and immune homeostasis within the ‘gut-liver axis’ incur a high risk for the development of these diseases and also for other major features of the metabolic syndrome (mainly cardiovascular complications like atherosclerosis and myocardial ischemia/infarction). In addition to nutritional imbalances, these immunometabolic diseases predispose gut-residing innate and adaptive immune cells to further alterations. Here, the innate branch of immune system plays a critical role in determining a proper balance between intestinal and low grade hepatic inflammation response(s).

Recently, plant seeds protease (serine-type) inhibitors (PPIs) have received much attention because of their proven implication, whether said implication worsens or improves, inflammatory and immune responses within the gut-liver axis [3,4]. PPIs are commonly found in the salt soluble fraction of seeds as part of protein complexes where monomeric units (~12–16 kDa, MW) display an immunonutritonal and therapeutic potential [5,6]. These proteins exhibit a highly disulfide-linked structure, which makes them partially resistant to gastrointestinal enzymes. This conformational disposition has been suggested to be responsible for the interaction of wheat PPIs with the innate immune ‘Toll-like’ receptor (TLR)-4/MD2/CD14 complex [3] of myeloid cells at intestinal level [7] Recent research identified protease (trypsin) inhibitory capacity in *C. quinoa* and *S. hispanica* [8,9], which was further supported by the presence of protease inhibitory (pancreatin) complexes in those seeds [6] Relatively high concentration of albumins and globulins in both *C. quinoa* [10,11] and *S. hispanica* L. have been described [12] However, the immunonutritional potential of these compounds remains to be elucidated. Notably, administration of a salt soluble extract from *C. quinoa* and *S. hispanica* enriched in PPIs to hepatocarcinoma (HCC) developing mice promoted immunonutritonal effects reducing liver injury. Animals fed with PPIs displayed significantly increased numbers of F4/80+ and CD68+ cells suggesting a significant role for PPIs in macrophage functional differentiation [4,6].

Macrophages (Mθ) represent a major cell type of innate immunity and have emerged as a critical player and therapeutic target in liver-associated immunonutritonal diseases [13]. The relevance of interventions aimed at favoring, in a selective way, a defined phenotypic differentiation of Mθ in the control of NAFLD and the HCC [13] has been previously highlighted. A means to selectively drive Mθ activation towards a defined functional differentiation (i.e., inflammatory/antitumoral, M1 or anti-inflammatory/protumoral M2 phenotype) can set the stage for a new generation of immunonutritional interventions based on their activity. However, the functional roles of PPIs in the macrophages’ metabolic and phenotypic adaptation remain to be elucidated. Advances in understanding how regulation of functional differentiation of macrophages capable of influencing immune responses can be modulated by immunonutritional compounds, could represent a path towards developing durable and long-lasting immune response(s).

This study aims to explore the immunonutritonal impact of a bioaccessible fraction from *C. quinoa* and *S. hispanica* on immune, metabolic and phenotypic changes in human-like macrophages, in order to better understand dietary interactions that can determine their cellular functional differentiation and programing.

## 2. Material and Methods

### 2.1. Cell Culture

Human-like macrophages (HB-8902^®^) were purchased from the ATCC (Mannasas, VA, USA). Cells were grown in 150 cm^2^ flasks in Eagle’s Minimum Essential Medium (EMEM) according to recommendations from ATCC. Cells cultures were kept in an incubator (37 °C/ 5%, *v/v*, CO_2_) and culture media were changed every two days. Prior to being used in the experiments, cells were seeded (5 × 104 cells cm^−2^ with 1 mL of EMEM) onto 12-well plates and returned to the incubator for an additional 24 h.

### 2.2. Isolation of Protease Inhibitors (PPIs) and Cell Culture Treatments

PPIs to be tested in cell cultures were obtained from seeds of *C. quinoa* and *S. hispanica* [6] obtained from local supermarkets. The protein concentration of the extracts (<30 KDa) was quantified in order to normalize the contents cell cultures were exposed to. Working solutions in EMEM were added to the cells to reach a final concentration of 100 µg·mL^−1^ (0.56% AU protease inhibitory activity) and incubated for 4 h. This concentration was established as effective at modulating immunonutritonal parameters in a preclinical model of severe liver dysfunction [4]. Control cells were used throughout exposed to either bovine serum albumin (heat shock fraction) (A7906, Sigma-Aldrich, Madrid, Spain), CM3 (A1520, Sigma-Aldrich) or bacterial lipopolysaccharide (LPS from *E. coli*) (L3129, Sigma-Aldrich). The assays were performed in two different days (n = 4).

### 2.3. FACS Analysis for TLR4 Expression and Macrophage Phenotypic Differentiation

HB8902^®^ cells incubated with PPIs (3 h) were collected with a cell scraper in EMEM. According to the manufacturer’s instructions, 1 µL of anti-human TLR4 (CD274, Cat. Nº 145404, BD Biosciences, San José, CA, USA), CD68 (Cat. Nº. 562117, BD Biosciences) and CD206 (Cat. Nº 555954, BD Pharmingen, San José, CA, USA) antibodies were added to cell suspensions. After gentle vortexing, samples were incubated for 15 min in the dark. Cells were then analyzed using the FACS Diva software (BD Biosciences). At least 10,000 events were analyzed for each independent sample.

### 2.4. Cell ‘Mito’ Stress Test Assay

Human-like macrophage (HB-8902^®^) cells were obtained from the ATCC and grown according to the recommended conditions. 24 h before the experiments, cells cultures were plated in a Seahorse 96 well plate (4 × 104 cells/well) and returned to the incubator. An extracellular flux analyzer (Seahorse Biosciences, Madrid, Spain) was used to assess the oxygen consumption rate (OCR) according to the manufacturer’s recommendation (Cat. No. 103015-100, Agilent, Madrid, Spain). Basal respiration was initially measured before the injection of oligomycin (2 µM) to determine ATP-linked OCR. Then maximal respiration was estimated by the addition of (4-trifluoromethoxycarbonyl cyanide phenylhydrazone (FCCP, 1.5 µM). Afterwards, a mixture with rotenone (0.5 µM) and antimycin A (0.5 µM) was used to determine the mitochondrial-independent OCR in order to correct all previously obtained measurements.

### 2.5. Measurement of Lysosomal (Neutral Red Assay) and Mitochondrial Enzyme (Test MTT) Activities

In HB8902^®^ cells, the influence of isolated PPIs on neutral red (TOX4, Sigma-Aldrich) and MTT (3-(4,5-dimethylthiazol-2-yl)-2,3-diphenyltetrazolium bromide) conversion (TOX1, Sigma-Aldrich) was measured using the aforementioned commercial kits. All data were referred to those obtained in untreated control cells.

### 2.6. Phospholipid Assay

Cells cultures were homogenized with the RIPA buffer (0.2 mL) and the total phospholipids content was quantified in an aliquot of this homogenate using a commercial ELISA kit (ab178622, Abcam, Cambridge, UK). Total protein content (Pierce^®^-BCA, Thermo Scientific™, Waltham, MA, USA) in the extract was used to normalize the phospholipid content in all samples.

### 2.7. Relative Gene Expression Analyses

Total RNA was extracted from HB-8902^®^ cells using RNeasy mini kit (Qiagen, Venlo, The Netherlands) according to the manufacturer’s instructions. Total RNA (500 ng) was converted to double-stranded cDNA using AMV Reverse Transcriptase (Promega, Madison, WI, USA). PCR was performed with primers designed (www.ncbi.nlm.nih.gov) for the following *Homo sapiens* genes: TLR4 (forward 5′-TAC TGC ACA AGG TGA GGT GTT-3′, reverse 5′-TGT CTC AGC CAA CTG CCT AC-3′), CD36 (forward 5′-AAA GGA CCC CTA GAG TCG CA-3′, reverse 5′-ACA GAC AGG CTC CAA GGA ATG-3′), angiopoietin-like 4 protein (forward 5′-CCT GCC TTC AAC CCC ACA TT-3′, reverse 5′-GAT GGG AAA ACT GAG GCC AGA-3′), and GAPDH (forward 5′-CCA CTC CTC CAC CTT TGA CG-3′; reverse 5′-CGC CAG ACC CTG CAC TTT TT-3′).

The PCR mixture (20 µL) [2.5 µL of cDNA, 10 µL of SybR Green buffer (Applied Biosystems, Life Technologies S.A., Madrid, Spain) and 0.5 µL of each primer] was amplified according to the following PCR program: 1 cycle of denaturation at 95 °C for 10 min, 35 cycles of amplification at 95 °C for 15 s, 60 °C for 20 s, and 72 °C for 30 s using a QuantStudio Real-Time PCR System (Applied Biosystems) system. The relative mRNA expression of the tested gene to the housekeeping was calculated using the 2^−ΔCp^ method.

### 2.8. Cell Cultures Total Protein Extraction

After the incubation period, cell culture supernatants were removed (×3) with phosphate-buffered saline (0.1 M, pH 7.2). Then, cellular extracts were obtained with 0.2 mL of RIPA buffer supplemented with protease inhibitors (Complete 04 693 116 001, Roche, Basel, Switzerland) [14]. Samples were centrifuged (8000× *g*/15 min/4 °C) to obtain clear supernatants to be used in proteome analyses.

### 2.9. In-Gel Digestion

Cell extracts were run in a conventional SDS-PAGE electrophoresis to concentrate the whole proteome in the stacking/resolving gel interface. The gel was stained with Coomassie and selected bands were placed in microcentrifuge tubes [15]. Proteins selected for analysis were in-gel reduced, alkylated, and digested with trypsin (Promega) as described elsewhere [16] with minor modifications. Briefly, spots were washed twice with water, shrunken for 15 min with acetonitrile 50% (*v/v*), and dried in a vacuum centrifuge for 30 min. After reduction with 10 mM DTT in 25 mM NH_4_HCO_3_ for 30 min at 55 °C, the samples were alkylated with 55 mM iodoacetamide in 50 mM NH_4_HCO_3_ in acetonitrile 50% (*v/v*) for 20 min. Then, samples were dehydrated with acetonitrile 100% for 5 min and digested with 60 ng·µL^−1^ sequencing grade trypsin (Promega) at 5:1 protein:trypsin (*w/w*) ratio for at least 12 h at 37 °C. Trifluoroacetic (TFA) acid (1% *v/v*) was used to stop the digestion and samples were desalted (OMIX Pipette tips C18, Agilent Technologies) prior to the analysis.

### 2.10. Reverse Phase-Liquid Chromatography RP-LC-MS/MS Analysis

The dried samples were dissolved in 0.1% formic acid to be analyzed in an Easy-nLC II system coupled to an ion trap LTQ-Orbitrap-Velos-Pro hybrid mass spectrometer (Thermo Scientific) [17].

The detection was performed with the following experimental conditions: survey scans from 400 to 1600 amu (1 μscan), twenty data dependent MS/MS scans, isolation width of 2u (in mass-to-charge ratio units), normalized collision energy of 35%, and dynamic exclusion applied for 30 s periods. Peptide identification from raw data was carried out using the SEQUEST algorithm (Proteome Discoverer 1.4, Thermo Scientific). A database search was performed against uniprot-Homo.fasta, uniprot-Bos.fasta and uniprot-Homo-Bos.fasta.

### 2.11. Statistical Analysis

The multivariate analysis of principal components was performed with Statgraphics© Plus (version 5.1, Rockville, MD, USA) [17]. Statistical significance for protein expression was set at *p* < 0.05 by applying one-way ANOVA and Tukey’s *post hoc* test. The Student’s *t* test was used to compare of the expression levels for the identified cell proteins.

## 3. Results and Discussion

### 3.1. Protein Patterns

**Figure 1:**
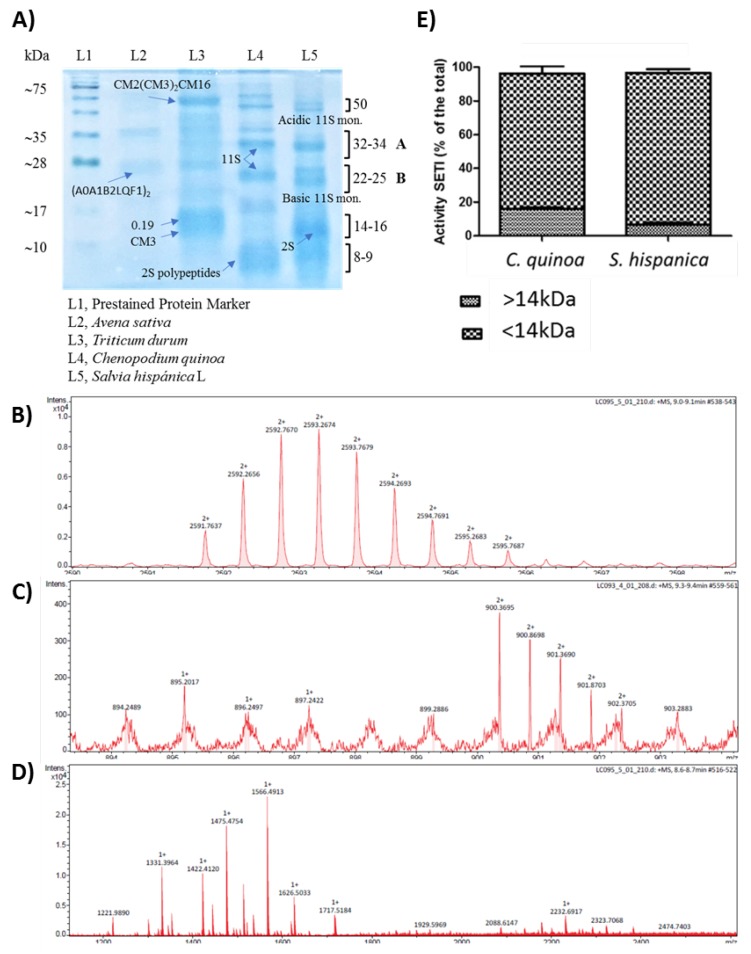
Electrophoretic (SDS-PAGE, 12%) pattern of salt soluble (pH 6) proteins from C. quinoa and *S. hispanica* (**A**), 11S storage protein that scaped postsynthetic processing (**B**), polypeptides from 2S-type proteins (**C**), oilseed 2S albumin (**D**), relative protease inhibitory activity in the <30 kDa fraction obtained from *C. quinoa* or *S. hispanica* L. (**E**).

Electrophoretic (SDS-PAGE) analysis of protein extracted from both crops (Figure 1) demonstrated that the patterns and relative abundance of polypeptides were nearly identical, indicating that all major albumin- and globulin-type proteins were represented under optimal extraction conditions (Figure 1A). 

The acidification at pH 6 of the extraction buffer allowed the preferential solubilization of 11S and 2S proteins. Increasing salt concentrations did not improve protein yield (*data not shown*). The corresponding bands to 11S globulin (chenopodin) [10,11] from *C. quinoa* are indicated in the figure by the letters A and B. The ~50 kDa band corresponds to the non-processed proglobulin with structural characteristics akin to 11S globulins, and those groups of ~8–9 kDa are similar to polypeptides [10,11] from 2S-type proteins. The ~14 kDa band in *S. hispanica* is similar in size to the ‘high-cysteine’ 2S albumin found in many oilseeds [18]. The electrophoretic pattern of *S. hispanica* also showed typical acidic (30 kDa) and basic (20 kDa) subunits from the 11S proteins found in chia seeds [12]. The RP-LC-MS/MS analysis was used to confirm these results. The homology between electrophoretic patterns obtained from both samples was further supported by the identification of ‘*m/z*’ signals corresponding to chenopodin (11S) that escaped postsynthetic processing (Figure 1B) as well as polypeptides from 2S-type proteins (Figure 1C) and the 2S albumin in oilseeds (Figure 1D). The study of serine-type protease inhibitors found in *Triticum durum* and *Avena sativa* is outside the scope of this study and was conducted as a positive control of the isolation process to check extraction conditions.

It was found that *C. quinoa* and *S. hispanica* L. provide partially resistant PPIs, in the dialyzable fraction obtained by a simulated human gastrointestinal digestion [6] (pepsin at pH 3, pancreatin-bile at pH 6) in a bicameral system. (Figure 1E). The elucidation of structural features allowed to identify these PPIs as glycoproteins with an *N*-terminal glucuronamide linkage in *S. hispanica* and glucosides in *C. quinoa* [6]. The RP-LC-MS/MS analyses confirmed the absence of LPS contamination during obtention of the bioaccessible fraction from simulated gastrointestinal digestion in order to evaluate their immunonutritional influence in the macrophage population.

### 3.2. Macrophage Polarization

**Figure 2:**
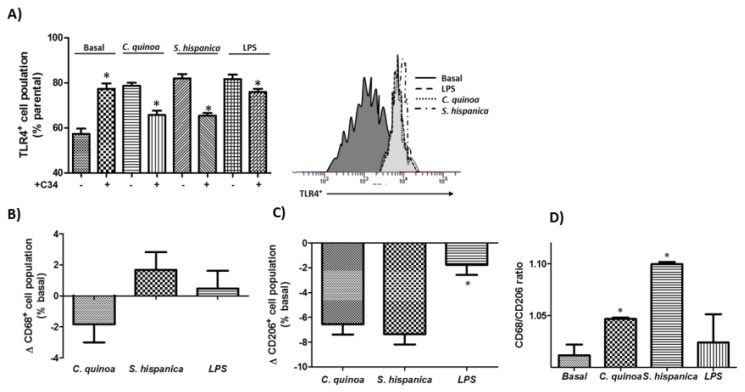
Identification of Toll-like receptor (TLR)-4 agonists. FACS analysis of TLR4 expression (**A**) and immunephenotyping of the macrophage population (**B**,**C**) challenged to protease inhibitors from *C. quinoa* or *S. hispanica* L. and lipopolysaccharide (10 ng/mL). (**D**) Variations of the CD68/CD206 ratio in HB8902 cultures. The results are expressed as mean ± standard deviation (n = 4). * Indicates statistical differences in relation to their counterparts.

HB8902^©^ cells were challenged (100 µg·mL^−1^) to the < 30 kDa fraction obtained from *C. quinoa* and *S. hispanica* with similar protease inhibitory potential: *C. quinoa*, 0.59 AU·mg^−1^ protein and *S. hispanica*, 0.52·AU·mg^−1^ protein (Figure 2). Both samples caused a significant up-regulation of TLR4 expression (Figure 2B), which was normalized to control culture values where cell cultures were pretreated with the C34 inhibitor. However, most significant differences between the immunephenotypic-induced effects originated in their opposite impact on the expression of CD68 biomarker (Figure 2C). Besides, it was quantified a similar reduction in the expression of CD206 was quantified (Figure 2D). In accordance to their interaction with TLR4, cell cultures displayed a positive CD68/CD206 ratio (Figure 2E). Collectively, these results show that PPIs exert immunomodulatory effects on macrophage population and can have a substantial effect on their functional differentiation towards a M1 phenotype. This phenotypic orientation is similar to the significant increase of M1 resident macrophages found in the small intestine [4] and liver [7] of animals fed with PPIs.

### 3.3. Immunonutritonal Changes

**Figure 3:**
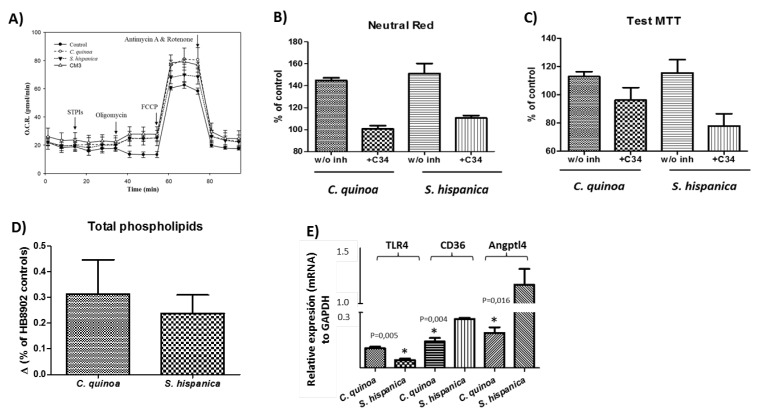
Metabolic changes in HB8902^©^ cells incubated (3 h) with extracts from *C. quinoa* or *S. hispanica* L. (**A**) Typical profiles of changes in oxygen consumption rates (OCR, pmol O_2_/min) (n = 6). (**B**) neutral red uptake due to endo/lysosomal activities (n = 4). (**C**) MTT conversion by mitochondrial dehydrogenases (n = 4). (**D**) Serine-type protease inhibitors-induced changes on total phospholipids content in human-like macrophages (HB8902) cells (n = 4). (**E**) Differential gene expression (mRNA) of Toll-like receptor 4 (TLR4), translocase of fatty acids (CD36) and angiopoietin-like 4 protein (Angptl4) (n = 4). * Indicates statistically significant (*p* < 0.05) differences in relation to its counterpart.

Macrophage metabolism is closely related to their functional differentiation state. Thus, taking advantage of the Seahorse^®^ technology cellular respiratory assay was performed to assess the mitochondrial metabolic orientation of cell cultures exposed to TLR4 agonists obtained from *C. quinoa* and *S. hispanica* (Figure 3).

The resulting energy map showed that macrophages challenged to the extracts exhibited increased oxygen consumption rates (OCR, pmol min^−1^) compared to controls (Figure 3A). Extracts from *C. quinoa* significantly increased spare respiratory capacity (60.2 ± 6.0 pmol·O_2_·min^−1^) compared to those incubated with extracts from *S. hispanica* L. (49.8 ± 4.2 pmol·O_2_·min^−1^) and controls (45.0 ± 7.2 pmol·O_2_·min^−1^). All cell cultures exhibited lower proton leak (*C. quinoa*, 2.2 ± 0.6 pmol O_2_ min^−1^ and *S. hispanica* L., 2.1 ± 0.8 pmol O_2_ min^−1^) than controls (4.4 ± 1.6 pmol·O_2_·min^−1^) confirming negligible deleterious effects in mitochondrial function. CM3, a PPI from wheat [3] served as a functional positive control, causing a slight positive variation (by 5.4%) of spare respiratory capacity. These changes were accompanied by increases in non-mitochondrial respiration in cultures challenged with *C. quinoa* (by 36.6%) and *S. hispanica* L. (by 28.0%) in relation to controls. The events observed in OCR mimic those of CM3 (by 15.1%) and are consistent with the inhibited mitochondrial oxidative respiration occurring during classical activation (i.e., LPS/IFN-γ) of macrophages [19]. Mitochondrial-independent oxygen consumption could be attributed to an increase in the activity of mono- and dioxygenases, which can be introduced by TLRs ligands such as LPS and play key regulatory roles in the immunomodulatory effects during chronic inflammation [20]. However, the lack of deleterious effects on mitochondrial function also allow us to hypothesize that PPIs do not suppress β-oxidation allowing lipid management by macrophages.

Numerous studies indicate that targeting macrophages to drive a selective functional differentiation of their phenotype may alleviate immunosuppression [4,13]. Thus, additional studies were performed to better understand the TLR4-induced metabolic adaptation response(s) by PPIs on the endo/lysosomal and mitochondrial compartment (Figure 3B,C). PPIs induced metabolic effects engaging both the mitochondrial and endo/lysosomal compartment. Said interaction, seemed to depend on TLR4 since pretreatment of cell cultures with C34 blunted PPIs-induced changes in the activity of these compartments. Endocytic retrieval of TLR4 is mediated by intracellular pathways (i.e., MCP-1 and IL-8) that are activated by phospholipids (PLs) [21]. To elucidate the potential of PPIs to induce changes on PLs content in HB8902 cells, the total PL content in cells cultures challenged to PPIs was quantified (Figure 3D). These observations suggest that PL can play important biological activities for macrophages in an environment where there is ‘sterile inflammation’ that may further drive immunonutritonal processes and macrophage function [4,6].

Additional analyses on the cellular transcriptome were performed to confirm the changes in the mRNA expression of TLR4 (Figure 3E). Also, changes in the transcripts of CD36 (translocase of fatty acids) and Angptl4 (angiopoietin-like protein 4) were determined due to their contribution to macrophage activation and anti/lipotoxic roles. The expression of CD36 and TLR4 decreased after PPI stimulation, in accordance with the TLR4 ligand recognition guiding PPIs to intracellular compartments. Also, this response is consistent with a continuous rather than transient activation of TLR4 [22] and CD36 expression, which has become increasingly associated to an M2 polarization [23]. Transcripts for Angptl4 showed opposite variations between cultures exposed to extracts from *C. quinoa* or *S. hispanica*. Previous studies have shown that Angptl4 expression is inhibited in activated macrophages [24]. The downregulation of Angptl4 mRNA expression is associated with increased protein translation and inhibition of lipid internalization. These changes go hand in hand with the differential expression of dihydrolipoyl dehydrogenase (P09622), which participates in lipoic acid metabolism, further influencing innate immunity into tissues [25].

### 3.4. Phenotypic Changes

**Figure 4:**
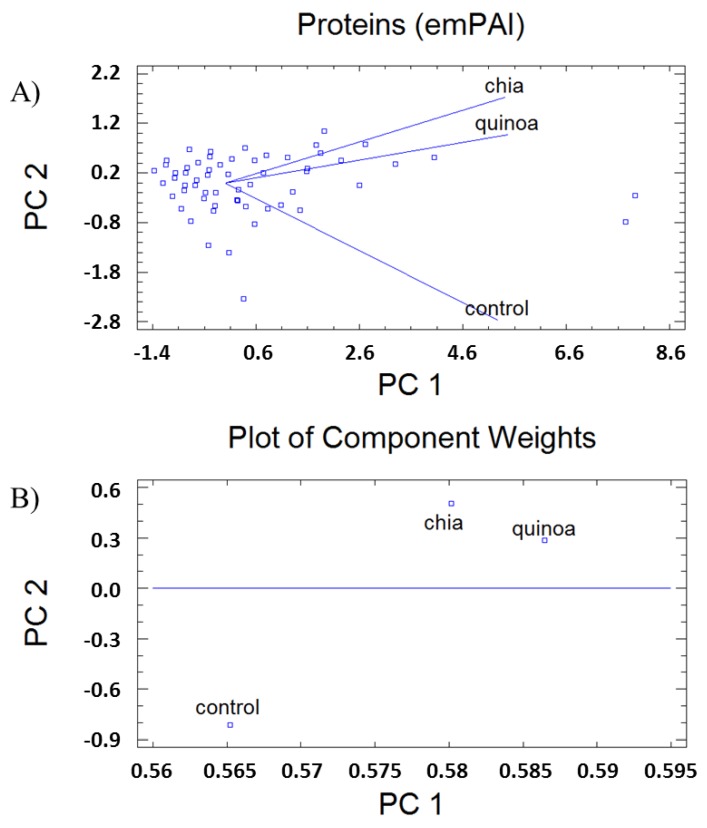
Principal component (PC) analysis (**A**) and plot of coefficients representing component weights (correlation coefficients between the studied factor and variables) (**B**) of changes in the proteome of human-like macrophage cells incubated with extracts from *Chenopodium quinoa* (quinoa) or *Salvia hispanica* L. (chia) in comparison to untreated control cultures.

**Figure 5:**
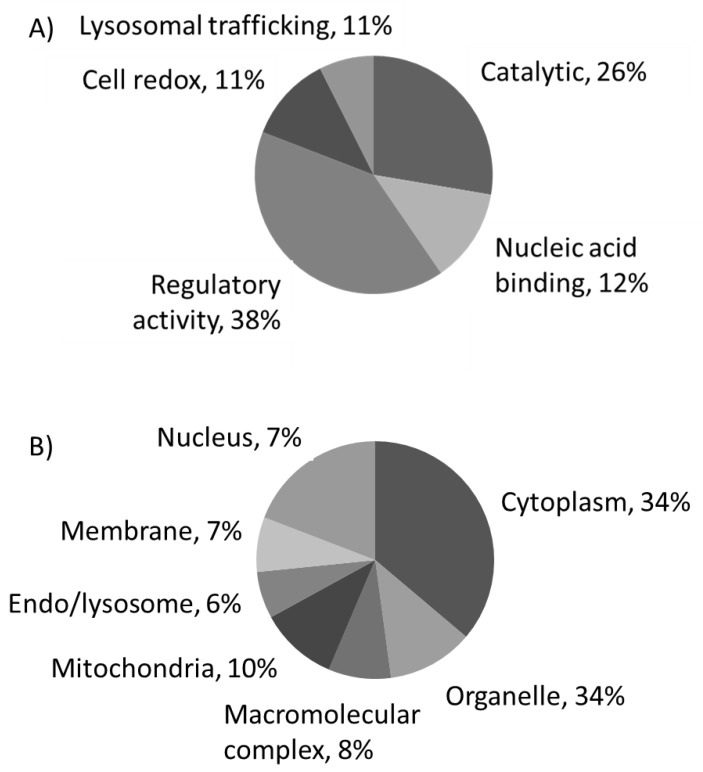
Summarized functional categorization of the positively identified human-like macrophage cell proteins, according to their molecular function (**A**) and their cellular localization (**B**).

Proteome analyses to reveal biological patterns triggered by immunonutritonal nutrients in HB-8902^®^ cells (Figure 4) were performed. The results revealed responses that could be grouped into different general function categories and subcellular distribution. Proteins were plotted against a gene ontology database to generate an overview of the regulatory and catalytic functions (Figure 4A), which were found at cytoplasm or organelle-associated compartments (Figure 4B). A principal component analysis allowed us to define the changes corresponding with the major weight of PC1 (Eigenvalue, 2.63) (Figure 5). In this component, it was identified proteins involved in glucose homeostasis, signal transduction, regulation of apoptosis, cell adhesion, cytoskeleton, down-regulation of TGF-β activation, modulation of type-I interferon mediated response(s) and telomere maintenance, MyD88/TRIF, MHC-I/II and IL-12/23 signaling were identified. The PC2 (Eigenvalue, 0.24) was associated with proteins involved in the transfer of phosphate groups, cellular response to glucose stimulus, phospholipid turnover, degradation of hypoxia inducible factor (HIF)-1α and stabilization of activated protein kinase C. The positively identified proteins are shown in Table 1.

Among the most relevant adaptation responses in the scope of this study, were changes in the expression levels of proteins involved in the glycolytic metabolism. The latter, favored a rapid production of energetic and reducing equivalents as well as increased levels of Krebs cycle intermediates, in concordance with the variations in OCR (pmol min^−1^) (Figure 3A), supporting an increased non-mitochondrial respiration. The variation of key mediators in glycolysis—P04406, P00558, P18669, P14618, O75874 and P40925—indicate an adaptation towards low utilization of carbohydrates together with the accumulation of tricarboxylic acid cycle intermediates (i.e., citrate, malate) favoring a classical and innate (i.e., TLRs)-induced M1 phenotype [21,26]. Otherwise, macrophage activation through the alternative pathway engaged modest metabolic events. Likewise, the reduced glycolytic metabolism is consistent with a cellular response to attenuate TLR4 signaling [23] (Figure 3C). The metabolic control also affected to the response to glucose uptake (Q13243-3, Q13247-3), thereby reflecting changes in cellular activity because of an oxygen-containing compound stimulus. The latter is concordant with the down-regulated expression of hypoxia up-regulated protein (Q9Y4L1), which contributes to reprogram lipid catabolism [27]. These changes seem to highlight a metabolic orientation skewing towrads oxidative metabolism.

The cellular response and association between high mobility group protein B1 (HMGB1) (P09429), (TGF)-β1 [28] and peroxisome proliferator-activated receptor (PPAR)-γ [29] occur in an environment (P63151, P0CG48, Q9Y263) dominated by TLR4 signaling towards downstream MyD88/TRIF signaling [30]. However, these biological responses do not appear to mediate mitochondrial dysfunction (O95831-3, P08758, P39656 and P04843) as LPS does.

At subcellular level, endosomal replenishment of the TLR4-MD2/CD14 complex is critical for controlling the breadth of signaling, and thereby the magnitude of innate immune response(s). The pattern in expression levels of different Rab proteins (P20340, P51149, Q15907, P61106) supports variations for the impact in late endosome membrane trafficking [31,32]. The differences are extended to the expression levels of Rab10 (P61026), likely influencing TLR4′s replenishment onto the plasma membrane [33], allowing us to assume a differential TLR4 agonistic capacity for the PPIs. Expression of different sets of mannose-receptors (P04843, P39656 and P04844) may thus confer antigen-type and intracellular-processing specificity [34] to different phenotypic macrophage subsets. Also, influences on molecular changes modulating endosomal acidification (P38606) transpire into differences in the engagement of adaptor molecules to activate MyD88-dependent signaling [35].

**Figure 6:**
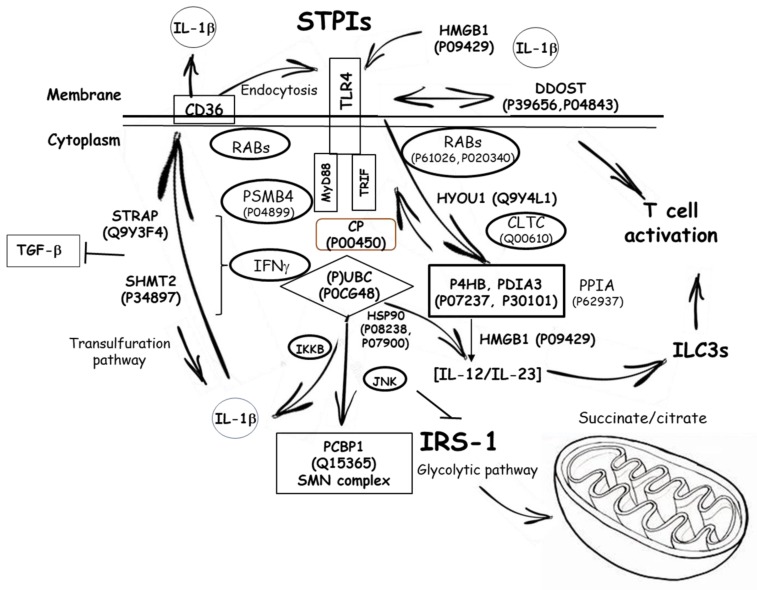
Proposed scheme of the interrelationship between the proteins with significant expression and the innate immune ‘Toll-like’ receptor (TLR)-4 during serine-type protease inhibitors stimulation of human-like macrophage cells. Abbreviations can be found in Table 1.

Activation of HB-8902^©^ by PPIs also promotes molecular changes—P63151, P61088, P08238, P04899, Q15185—in ‘Toll interacting proteins’ [36,37] and downstream effectors of TLR4-associated molecules such as CD36 and members of it’s signaling pathway [38]. These contribute to the negative regulation of TGF-β1-mediated activation of Smad signaling pathways [28] and the endogenous production of anti-inflammatory agents modulating TLR4 linked cytokine synthesis [39]. Altogether, it could be hypothesized that PPIs might favor a certain ‘tolerance’ to TLR4-driven signals, that in turn modulate macrophage physiological function. PPIs triggered metabolic forces and cellular processes associated to downstream TLR4 signaling that can be schematically depicted as shown in Figure 6.

Overall, these results provide further details into the cellular response(s) of human-like macrophage (HB-8902^©^) cells to TLR4-mediated immunonutritonal effects by PPIs from *C. quinoa* or *S. hispanica*, and how these are associated to macrophage functional differentiation. Currently, despite the known relative high proportion of PPIs in *C. quinoa* and *S. hispanica* it is difficult to find studies at systems biology level with a grander focus beyond its nutritional value. Notably, the scarce existing in vivo studies have shown their positive potential improving immunonutritonal control of dietary PPIs in HCC developing mice contributing to develop a phenotype with reduced tumor burden in preclinical models [4,6]. These positive effects warrant further research.

## 4. Conclusions

Immunometabolic effects of bioaccesible polypeptides provided by 2S albumin from *C. quinoa* and *S. hispanica* L. cause cellular response(s) associated to TLR4 signaling driving the functional differentiation of human-like macrophages. The observed immunometabolic responses argue in favor of beneficial effects of PPIs in selective macrophage functional differentiation, as observed by others, towards a M1 phenotype. Here, the potential immunometabolic effects triggered by protein fractions from *C. quinoa* and *S. hispanica* can help to tailor dietary ‘trained immunity’ intervention strategies in immunometabolic (i.e., obesity, hepatocarcinoma) based diseases. Molecular-level understanding of immune-metabolic associations enable a better grasp of the role of immunonutritional components in influencing innate immune response(s) to reduce immune imbalances and, thereby the risk of metabolic diseases. These potential benefits warrant further research to approach immunonutritional compounds for the prevention and management of diet-associated innate immune imbalances where physiological and genetic characteristics can have important effects.

## Figures and Tables

**Table 1:** Proteins identified by RP-LC-MS/MS in human-like macrophage cells incubated with extracts from *Chenopodium quinoa* and *Salvia hispanica* L. compared to untreated controls.

Nº (#)	Protein ^a^ (Accession)	Mr	pI	Mean Fold Difference ^b^		*p* ^c^
				*C. quinoa*	*S. hispanica* L.	
1	Heat shock protein HSP 90-beta (P08238)	83.2	5.03	1.23 ± 0.28	0.89 ± 0.28	0.103
2	Pyruvate kinase (P14618)	57.9	7.84	0.13 ± 0.03 *	0.11 ± 0.03 *	0.446
3	Malate dehydrogenase (P40926)	35.5	8.68	1.92 ± 0.44 *	1.66 ± 0.53	0.444
4	Polyubiquitin-C (P0CG48)	77.0	7.66	1.34 ± 0.31	1.16 ± 0.37	0.445
5	Transitional endoplasmic reticulum ATPase (P55072)	89.3	5.26	1.40 ± 0.32	1.22 ± 0.39	0.443
6	T-complex protein 1 subunit zeta (P40227)	58.0	6.68	0.24 ± 0.05 *	0.21 ± 0.07 *	0.446
7	Protein disulfide-isomerase (P07237)	57.1	4.87	1.30 ± 0.30	0.97 ± 0.31	0.124
8	Peptidyl-prolyl *cis-trans* isomerase A (P62937)	18.0	7.81	1.71 ± 0.38	1.25 ± 0.40	0.124
9	6-phosphogluconate dehydrogenase (P52209-2)	51.8	7.44	1.61 ± 0.38	1.46 ± 0.46	0.445
10	Ras-related protein Rab-7a (P51149)	23.5	6.70	0.96 ± 0.22	0.62 ± 0.20	0.037
11	Aldehyde dehydrogenase (P05091-2)	51.0	6.20	0.28 ± 0.06 *	0.97 ± 0.31	0.001
12	D-3-phosphoglycerate dehydrogenase (O43175)	56.6	6.71	1.68 ± 0.38	1.45 ± 0.47	0.445
13	L-lactate dehydrogenase B chain (P07195)	36.6	6.05	1.96 ± 0.45 *	0.97 ± 0.31	0.004
14	Sodium/potassium-transporting ATPase (P05023-3)	109.5	5.31	1.68 ± 0.38	0.36 ± 0.11 *	0.001
15	Fructose-bisphosphate aldolase A (P04075)	39.4	8.09	0.72 ± 0.16	1.45 ± 0.45	0.010
16	Annexin A5 (P08758)	35.9	5.05	2.01 ± 0.46 *	2.04 ± 0.65	0.932
17	Voltage-dependent anion-selective channel (P45880-2)	30.4	7.20	1.34 ± 0.31	1.16 ± 0.38	0.445
18	L-lactate dehydrogenase A chain (P00338)	36.7	8.27	1.34 ± 0.31	0.87 ± 0.28	0.037
19	Macrophage-capping protein (P40121-2)	36.8	7.20	1.67 ± 0.38	1.45 ± 0.46	0.446
20	Dolichyl-diphosphooligosaccharide-glycosyltransferase (P39656-3)	49.0	6.86	0.33 ± 0.88 *	0.29 ± 0.09 *	0.446
21	Nucleoside diphosphate kinase A (P15531)	17.1	6.19	1.68 ± 0.27	1.45 ± 0.46	0.445
22	T-complex protein 1 subunit delta (P50991)	57.9	7.83	1.20 ± 0.27	0.21 ± 0.07 *	0.001
23	Ubiquitin-like modifier-activating enzyme 1 (P22314-2)	113.7	5.60	0.67 ± 0.15	0.58 ± 0.17	0.446
24	Serine hydroxymethyl-transferase (P34897-3)	53.4	8.15	1.70 ± 0.38	1.42 ± 0.32	0.445
25	Malate dehydrogenase (P40925-2)	27.0	9.11	1.67 ± 0.38	1.54 ± 0.41	0.446
26	Serine/threonine kinase receptor-associated protein (Q9Y3F4)	38.4	5.12	0.42 ± 0.10 *	0.36 ± 0.11 *	0.446
27	Glucose-6-phosphate isomerase (P06744)	63.1	8.32	1.22 ± 0.25	0.97 ± 0.13	0.446
28	T-complex protein 1 subunit α (P17987)	60.3	6.11	1.12 ± 0.19	0.48 ± 0.15 *	0.001
29	Chloride intracellular channel protein 1 (O00299)	26.9	5.17	1.86 ± 0.34	1.39 ± 0.43	0.446
30	Nascent polypeptide-associated complex subunit α (Q13765)	23.4	4.56	1.12 ± 0.25	0.49 ± 0.15 *	0.002
31	Rho GDP-dissociation inhibitor 1 (P52565)	23.2	5.11	2.51 ± 0.57 *	1.54 ± 0.62	0.013
32	Glucose-6-phosphate 1-dehydrogenase (P11413)	59.2	6.84	1.00 ± 0.23	0.87 ± 0.27	0.446
33	Glyceraldehyde-3-phosphate dehydrogenase (P04406)	36.0	8.46	0.56 ± 0.13 *	0.48 ± 0.15 *	0.446
34	Dihydrolipoyl dehydrogenase (P09622-2)	43.6	7.03	1.67 ± 0.38	0.72 ± 0.23	0.002
35	Ras-related protein Rab-10 (P61026)	22.5	8.38	1.68 ± 0.38	0.97 ± 0.26	0.013
36	V-type proton ATPase catalytic subunit A (P38606-2)	64.7	5.66	1.12 ± 0.25	0.51 ± 0.15 *	0.002
37	Poly(rC)-binding protein 1 (Q15365)	37.5	7.09	2.24 ± 0.51 *	1.45 ± 0.46	0.037
38	Voltage-dependent anion-selective channel protein 1 (P21796)	30.8	8.54	1.26 ± 0.29	0.36 ± 0.11 *	0.001
39	T-complex protein 1 subunit ε (P48643-2)	49.5	6.19	1.55 ± 0.09	0.42 ± 0.12 *	0.001
40	Peptidyl-prolyl cis-trans isomerase FKBP4 (Q02790)	51.8	5.43	0.42 ± 0.10 *	0.38 ± 0.13 *	0.446
41	Ras-related protein Rab-1A (P62820)	22.7	6.21	0.56 ± 0.13 *	0.97 ± 0.31	0.025
42	T-complex protein 1 subunit eta(Q99832-3)	54.8	7.68	1.68 ± 0.38	0.49 ± 0.14 *	0.001
43	Ras-related protein Rab-2A (P61019-2)	20.8	6.11	1.67 ± 0.38	0.72 ± 0.23	0.002
44	Isocitrate dehydrogenase [NADP] cytoplasmic (O75874)	46.6	7.01	1.51 ± 0.33	1.42 ± 0.08	0.446
45	Isocitrate dehydrogenase [NADP], mitochondrial (P48735-2)	45.2	7.75	2.51 ± 0.57 *	0.72 ± 0.32	0.001
46	Fermitin family homolog 3 (Q86UX7-2)	75.4	6.77	0.56 ± 0.13 *	0.48 ± 0.13 *	0.446
47	Dolichyl-diphosphooligosaccharide--protein glycosyltransferase subunit 2 (P04844-2)	67.7	6.06	0.84 ± 0.19	0.79 ± 0.23	0.446
48	Receptor of activated protein C kinase 1 (P63244)	35.1	7.69	3.35 ± 0.77 *	1.56 ± 0.46	0.001
49	Ceruloplasmin (P00450)	122.1	5.72	2.51 ± 0.57 *	2.18 ± 0.70	0.446
50	Hypoxia up-regulated protein 1 (Q9Y4L1)	111.3	5.22	0.84 ± 0.19	0.76 ± 0.32	0.446
51	SRP55-3 of Serine/arginine-rich splicing factor 6 (Q13247-3)	38.4	11.00	1.67 ± 0.38	0.87 ± 0.41	0.002
52	High mobility group protein B1 (P09429)	24.9	5.74	0.84 ± 0.19	0.69 ± 0.17	0.446
53	Cell division control protein 42 homolog (P60953)	21.2	6.55	0.91 ± 0.12	1.67 ± 0.26	0.025
54	Ran GTPase-activating protein 1 (P46060)	63.5	4.68	1.69 ± 0.28	0.72 ± 0.17	0.002
55	NADPH-cytochrome P450 reductase (P16435)	76.6	5.58	1.75 ± 0.42	0.81 ± 0.22	0.002
56	Glutamate dehydrogenase 1 (P00367-3)	46.5	7.08	0.84 ± 0.19	0.76 ± 0.21	0.446
57	Ras GTPase-activating-like protein IQGAP1 (P46940)	189.1	6.48	0.84 ± 0.19	0.79 ± 0.18	0.446
58	Proliferation-associated protein 2G4 (Q9UQ80-2)	38.0	7.53	1.26 ± 0.29	0.54 ± 0.17	0.001
59	Protein disulfide-isomerase A3 (P30101)	56.7	6.35	1.33 ± 0.19	0.47 ± 0.09	0.001
60	GTP-binding nuclear protein Ran (P62826)	24.4	7.49	2.24 ± 0.51 *	1.94 ± 0.62	0.445
61	Calnexin (P27824)	67.5	4.60	0.89 ± 0.19	0.76 ± 0.32	0.446
62	Serine/threonine-protein phosphatase 2A 55 kDa regulatory subunit B (P63151)	51.7	6.20	0.85 ± 0.16	1.45 ± 0.34	0.025
63	Apoptosis-inducing factor 1 (O95831-3)	66.3	8.94	0.56 ± 0.13 *	0.48 ± 0.15 *	0.446
64	Phosphoglycerate kinase 1 (P00558)	44.6	8.10	1.92 ± 0.44 *	1.64 ± 0.36	0.148
65	Phosphoglycerate mutase 1 (P18669)	28.8	7.18	1.81 ± 0.38	1.25 ± 0.40	0.124
66	40S ribosomal protein S3 (P23396)	26.7	9.66	1.66 ± 0.28	1.13 ± 0.36	0.050
67	Rab GDP dissociation inhibitor β (P50395)	50.6	6.47	0.42 ± 0.10 *	0.36 ± 0.11 *	0.446
68	T-complex protein 1 subunit γ (P49368)	60.5	6.49	0.21 ± 0.05 *	1.09 ± 0.35	0.001
69	T-complex protein 1 subunit τ (P50990-3)	51.6	5.24	1.12 ± 0.25	0.48 ± 0.16 *	0.001
70	Serine/threonine-protein phosphatase PP1-α (P62136)	37.5	6.33	1.00 ± 0.23	0.29 ± 0.09 *	0.002
71	T-complex protein 1 subunit β (P78371-2)	52.7	6.44	2.10 ± 0.48 *	1.56 ± 0.36	0.066
72	Serine/threonine-protein phosphatase PP1-γ (P36873)	37.0	6.54	1.26 ± 0.29	0.37 ± 0.10 *	0.001
73	Peroxiredoxin-1 (Q06830)	22.1	8.13	1.20 ± 0.27	0.83 ± 0.26	0.066
74	Sarcoplasmic/endoplasmic reticulum calcium ATPase 2 (P16615-2)	109.6	5.36	1.12 ± 0.25	0.65 ± 0.18 *	0.002
75	Plasminogen activator inhibitor 1 RNA-binding protein (Q8NC51-4)	42.4	8.44	1.67 ± 0.38	1.74 ± 0.42	0.446
76	Proliferating cell nuclear antigen (P12004)	28.8	4.69	0.84 ± 0.19	0.72 ± 0.23	0.446
77	Proteasome subunit beta type-5 (P28074)	28.5	6.92	0.42 ± 0.10 *	0.18 ± 0.05 *	0.002
78	Splicing factor, proline- and glutamine-rich (P23246)	76.1	9.44	1.12 ± 0.25	1.78 ± 0.52	0.190
79	Protein disulfide-isomerase A4 (P13667)	72.9	5.07	1.68 ± 0.38	0.97 ± 0.26	0.013
80	Nucleosome assembly protein 1-like 1 (P55209-2)	42.7	4.55	0.56 ± 0.13 *	0.84 ± 0.16 *	0.446
81	Ubiquitin-conjugating enzyme E2 N (P61088)	17.1	6.57	0.84 ± 0.19	0.72 ± 0.23	0.446
82	Calcyclin-binding protein (Q9HB71)	26.2	8.25	0.96 ± 0.15	0.87 ± 0.32	0.446
83	SRP40-4 of Serine/arginine-rich splicing factor 5 (Q13243-3)	30.8	11.66	0.81 ± 0.14	0.76 ± 0.13	0.446
84	Prostaglandin E synthase 3 (Q15185-3)	14.9	4.77	0.80 ± 0.05	0.81 ± 0.09	0.446
85	V-type proton ATPase subunit C1 (P21283)	43.9	7.46	0.88 ± 0.19	0.72 ± 0.06	0.446
86	Heat shock protein HSP 90-α (P07900)	84.6	5.02	1.32 ± 0.30	0.99 ± 0.32	0.135
87	RuvB-like 1 (Q9Y265)	42.1	6.38	0.56 ± 0.13 *	0.48 ± 0.15 *	0.446
88	Phospholipase A-2-activating protein (Q9Y263)	50.2	6.42	1.67 ± 0.38	1.75 ± 0.27	0.446
89	Ras-related protein Rab-14 (P61106)	87.1	6.37	5.03 ± 1.15 *	2.91 ± 0.39 *	0.013
90	Prolyl endopeptidase (P48147)	23.9	6.21	1.75 ± 0.31	1.45 ± 0.44	0.446
91	V-type proton ATPase subunit B (P21281)	80.6	5.86	1.67 ± 0.42	1.42 ± 0.12	0.446
92	Ras-related protein Rab-6A (P20340)	56.5	5.81	1.57 ± 0.38	1.45 ± 0.08	0.446
93	Dolichyl-diphosphooligosaccharide-protein glycosyltransferase subunit 1 (P04843)	23.6	5.54	1.37 ± 0.36	2.87 ± 0.64 *	0.025
94	Mitochondrial import receptor subunit TOM40 homolog (O96008-2)	68.5	6.38	3.35 ± 0.76 *	1.28 ± 0.24	0.002
95	Ras-related protein Rab-11B (Q15907)	34.4	7.24	1.71 ± 0.38	1.54 ± 0.46	0.446
96	Serine/arginine-rich splicing factor 1 (Q07955)	24.5	5.94	1.69 ± 0.23	1.72 ± 0.31	0.446
97	Inter-α-trypsin inhibitor heavy chain H2 (P19823)	27.7	10.35	1.66 ± 0.35	2.91 ± 0.93 *	0.025
98	Ras GTPase-activating protein-binding protein 1 (Q13283)	106.3	6.85	1.59 ± 0.37	1.33 ± 0.46	0.446
99	Clathrin heavy chain 1 (Q00610-2)	187.8	5.69	1.68 ± 0.38	0.29 ± 0.09 *	0.001
100	Proteasome subunit beta type-4 (P28070)	29.2	5.97	0.84 ± 0.19	0.72 ± 0.23	0.446
101	Guanine nucleotide-binding protein G ‘α’ (P04899)	40.5	5.54	1.76 ± 0.26 *	1.45 ± 0.31	0.013

^a^ Molecular mass (Mr, Da) and Swissprot accession name; ^b^ The fold change of each protein is calculated as ratio between emPAI values, stress/control; emPAI = 10PAI - 1 where PAI is defined as PAI = N_obsd_/N_obsbl_ and N_obsd_ and N_obsbl_ are the number of observed peptides per protein and the number of observable peptides per protein, respectively; ^c^ *p* values calculated for statistical comparison between cell cultures exposed to extracts from *C. quinoa* or *S. hispanica* L; * Statistical (*p* < 0.05) comparison between control cultures and those exposed to extracts from *C. quinoa* or *S. hispanica* L.
